# Usefulness of mid-week hemoglobin measurement for anemia management in patients undergoing hemodialysis: a retrospective cohort study

**DOI:** 10.1186/s12882-019-1492-x

**Published:** 2019-08-02

**Authors:** Soo Ya Bae, Jae Wan Jeon, Seong Hoon Kim, Chung Hee Baek, Jai Won Jang, Won Seok Yang, Soon Bae Kim, Su-Kil Park, Sang Koo Lee, Hyosang Kim

**Affiliations:** 1 Department of Internal Medicine, Busan Bumin Hospital, Busan, Republic of Korea; 2 Division of Nephrology, Department of Internal Medicine, College of Medicine, Chungnam National University Hospital, Daejeon, Republic of Korea; 3 Department of Internal Medicine, Asan Medical Center, University of Ulsan College of Medicine, Seoul, Republic of Korea; 4 Division of Nephrology, Department of Internal Medicine, Asan Medical Center, University of Ulsan College of Medicine, 88, Olympic-ro 43-gil, Songpa-gu, Seoul 05505 Republic of Korea

**Keywords:** Hemoglobin, Anemia, Chronic kidney disease, Hemodialysis, Erythropoiesis-stimulating agent

## Abstract

**Background:**

Short-term hemoglobin (Hb) variability related to volume status is observed in chronic kidney disease (CKD) patients receiving hemodialysis (HD). Given the lack of studies regarding outcomes according to the day of Hb sampling, the existing guidelines do not strongly recommend regarding measurement timing. Pre-dialysis mid-week sampling (Wednesday and Thursday) is preferable to minimize short-term Hb variability, although numerous HD centers perform early-week sampling (Monday and Tuesday). The different measurement days may influence the prescribed dose of erythropoiesis-stimulating agent (ESA) and related patient outcomes. We investigated changes in Hb levels and ESA doses according to the Hb measurement day among HD patients.

**Methods:**

Starting September 2013, the day for pre-dialysis Hb measurement at the Asan Medical Center was changed from early-week days to mid-week days. This single-center retrospective study evaluated medical records of 92 patients who received maintenance HD between September 2012 and August 2014.

**Results:**

There was no significant difference in the mean Hb levels between early-week days and mid-week days (10.71 ± 0.06 g/dL vs. 10.78 ± 0.47 g/dL, *p* = 0.105). However, the mean doses of darbepoetin-α on early-week days were higher than those on mid-week days (175.4 ± 72.5 μg/month vs. 163.7 ± 83.6 μg/month, *p* = 0.022). The mean doses of intravenous iron hydroxide sucrose for early-week measurements were also higher than those for mid-week measurements (623.0 ± 489.0 mg/year vs. 447.0 ± 505.2 mg/year, *p* = 0.001). The mean interdialytic weight gains were 2.81 ± 0.82 kg on early-week days and 1.99 ± 0.61 kg on mid-week days (*p* < 0.001).

**Conclusions:**

Compared with early-week measurements, mid-week pre-dialysis Hb measurements were significantly associated with lower ESA doses without a change in Hb levels.

## Background

Anemia is a common feature of chronic kidney disease (CKD) and is related to poor outcomes, such as reduced quality of life, cardiovascular disease, and mortality [1]. Anemia in CKD patients is a multifactorial process associated with a relative erythropoietin (EPO) deficiency, uremic-induced inhibition of erythropoiesis, shortened erythrocyte survival, and disordered iron homeostasis [2]. The introduction of recombinant human EPO (rhEPO) and erythropoiesis-stimulating agents (ESAs) has helped limit anemia-related symptoms and reduce the need for repeated blood transfusions. However, ESA treatment has various adverse effects such as hypertension, seizure, and vascular access clotting [3, 4]. Recent randomized controlled trials have also indicated that correction of hemoglobin (Hb) levels to near the normal range was associated with increased risks of mortality, cardiovascular events, and stroke [5–12]. Secondary analysis of trial data also indicated that higher doses of ESA or ESA hypo-responsiveness were related to poor outcomes [11–16]. Therefore, the National Kidney Foundation Kidney Disease Outcomes Quality Initiative (KDOQI) guidelines recommend targeting Hb levels of 11.0–12.0 g/dL and not greater than 13.0 g/dL in CKD patients receiving dialysis or ESA therapy without dialysis [17]. The Kidney Disease Improving Global Outcomes (KDIGO) guidelines recommend judicious and individualized ESA therapy, although ESA treatment is generally not used to maintain an Hb level of > 11.5 g/dL [18].

The fluctuation in measured Hb levels in CKD patients (Hb variability) can be related to ESA therapy, iron status, and other CKD-related and -unrelated comorbid conditions [19, 20]. For example, hydration status significantly influences the short-term Hb variability, especially in patients with end-stage renal disease (ESRD) receiving hemodialysis (HD) [21–23]. Thus, there have been several discussions regarding determining a representative Hb value in ESRD patients, although these discussions have failed to produce a definitive conclusion [24–26]. Furthermore, the published guidelines for anemia do not firmly recommend regarding the timing of Hb measurement in ESRD patients receiving HD, given the lack of studies regarding patient outcomes according to the Hb measurement day [18, 27, 28]. Nevertheless, it is important to select optimal ESA dosing based on representative Hb levels and appropriate targets because unnecessarily low or high ESA doses are associated with poor outcomes. Therefore, we investigated the changes in Hb levels and ESA doses according to the Hb measurement day among ESRD patients receiving maintenance HD.

## Methods

### Study population

Starting September 2013, pre-dialysis Hb measurements were changed from early-week days (Monday or Tuesday, days after a long interdialytic period) to mid-week days (Wednesday or Thursday, days after a short interdialytic period) at the HD unit in Asan Medical Center (AMC), Seoul, South Korea. This single-center retrospective study evaluated medical records of patients receiving maintenance HD at the AMC HD unit between September 2012 and August 2014. Patients had conventional HD for four hours a day, three times a week. For patients who had dialysis for over five years, we used high flux membranes. There was no use of convective treatments nor high cutoff membranes. A total of 120 patients were identified who had anemia, were > 20 years old, were receiving maintenance HD for ≥3 months, and continued treatment at the AMC HD unit during the 2-year study period. Eight patients with massive bleeding events that required transfusions, 11 who underwent a major operation with considerable bleeding risk, 1 with an untreated gastrointestinal tract malignancy, 1 with a cytopenia event caused by treatment for chronic hepatitis C, 1 who was hospitalized for > 2 months, 3 with preexisting non-anemia hematological disorders, 2 who refused ESA treatment because of poor compliance, and 1 with a formal do-not-resuscitate order were excluded. Thus, the present study included 92 patients. The study protocol was approved by the institutional review board of AMC. Informed consent was not required, given the retrospective observational study design.

### Measurements

The baseline clinical characteristics of interest were age, sex, cause of ESRD, medical history, surgical history, and medication history. The laboratory parameters of interest were Hb, hematocrit, dialysis adequacy, albumin, prealbumin, high-sensitivity C-reactive protein, intact parathyroid hormone, ferritin, and transferrin saturation. These parameters were usually measured once a month except for prealbumin, dialysis adequacy, high-sensitive C-reactive protein, intact parathyroid hormone which were checked every 3 months. When Hb and Hct checked more than once a month for some reasons other than the exclusion criterion, we used the average value of them. The doses of ESAs and intravenous (IV) iron were also reviewed. The parameters of interest of the volume status were interdialytic body weight gain, ultrafiltration volume, and pre-dialysis body weight/dry body weight on the Hb measurement day. The ESAs used for patients were rhEPO-α (Epokine® in a prefilled syringe; CJ HealthCare, Seoul, South Korea) and darbepoetin-α (Nesp® in a prefilled syringe; Kyowa Hakko Kirin Co., Ltd., Tokyo, Japan). The weekly dose of rhEPO-α was divided by 200 to convert it into the corresponding weekly dose of darbepoetin-α. 200:1 is a conventional ratio used when weekly dose of rhEPO-α is converted to darbepoetin-α [29]. The IV iron was administered as Venoferrum® (JW Pharmaceutical Inc., Seoul, Korea), which contains ferric hydroxide sucrose complex (2700 mg) and Fe^3+^ (100 mg) in a single ampoule.

### Statistical analysis

The paired values from the early-week and mid-week measurements were compared. By Kolmogorov-Smirnov test, Hb, dose of ESA, ultrafiltration volume, Kt/V, prealbumin, iPTH were normally distributed. Parametric continuous variables were analyzed using the paired t test, and were reported as mean ± standard deviation or median (range or interquartile range), as appropriate. Non-parametric continuous variables were analyzed using Wilcoxon signed rank test. Differences in the numbers of patients who achieved the target Hb levels were evaluated using Pearson’s chi-square test and Fisher’s exact test. Differences in the ESA doses according to the achievement of the targeted Hb levels were evaluated using the Kruskal-Wallis test. All analyses were performed using IBM SPSS software (version 20; IBM Corp., Armonk, NY), and differences were considered statistically significant at *p*-values of < 0.05.

## Results

Table 1 shows the patients’ baseline characteristics. The mean age was 61.6 years, and diabetes mellitus was the leading cause of ESRD (50.0%). At baseline, some patients had histories of cardiovascular disease (23.9%), cerebrovascular accident (17.4%), malignancy (18.5%), kidney transplantation (9.8%), and other solid organ transplantation (3.3%). Most of the patients were receiving anti-hypertensive agents (93.5%) and antiplatelet agents (81.5%), while only a small proportion were receiving anticoagulant agents (4.3%).

**Table 1:** Baseline characteristics

	Values
Age (years)	61.6 ± 12.1
Sex	
Male	46 (50.0%)
Duration of HD (years)	8.3 ± 4.9
Cause of ESRD	
DM	46 (50.0%)
HTN	21 (22.8%)
Primary GN	8 (8.7%)
ADPKD	2 (2.2%)
Others	8 (8.7%)
Unknown	5 (5.4%)
Medical history	
DM	46 (50.0%)
HTN	86 (93.5%)
CVD	22 (23.9%)
CHF	12 (13.0%)
VHD	7 (7.6%)
CAOD	11 (12.0%)
PAOD	4 (4.3%)
Arrhythmia	6 (6.5%)
CVA	16 (17.4%)
Bleeding ^a^	10 (10.9%)
Chronic liver disease	9 (9.8%)
Malignancy	17 (18.5%)
Thyroid illness	8 (8.7%)
Medication history	
Antiplatelet agents	75 (81.5%)
Anticoagulants	4 (4.3%)
Antihypertensive agents	86 (93.5%)
Thyroid hormones	5 (5.4%)
Surgical history	
Parathyroidectomy	4 (4.3%)
Heart operation	7 (7.6%)
Kidney transplantation	9 (9.8%)
Solid organ transplantation ^b^	3 (3.3%)

Values are given as number (%) or mean ± standard deviation

^a^ Gastrointestinal tract bleeding, hemoptysis. ^b^ Other than the kidneys

DM, diabetes mellitus; HD, hemodialysis; HTN, hypertension; GN, glomerulonephropathy; ADPKD, autosomal dominant polycystic kidney disease; CVD, cardiovascular disease; CHF, congestive heart failure; VHD, valvular heart disease; CAOD, coronary artery occlusive disease; PAOD, peripheral artery occlusive disease; CVA, cerebrovascular accident

**Fig. 1:**
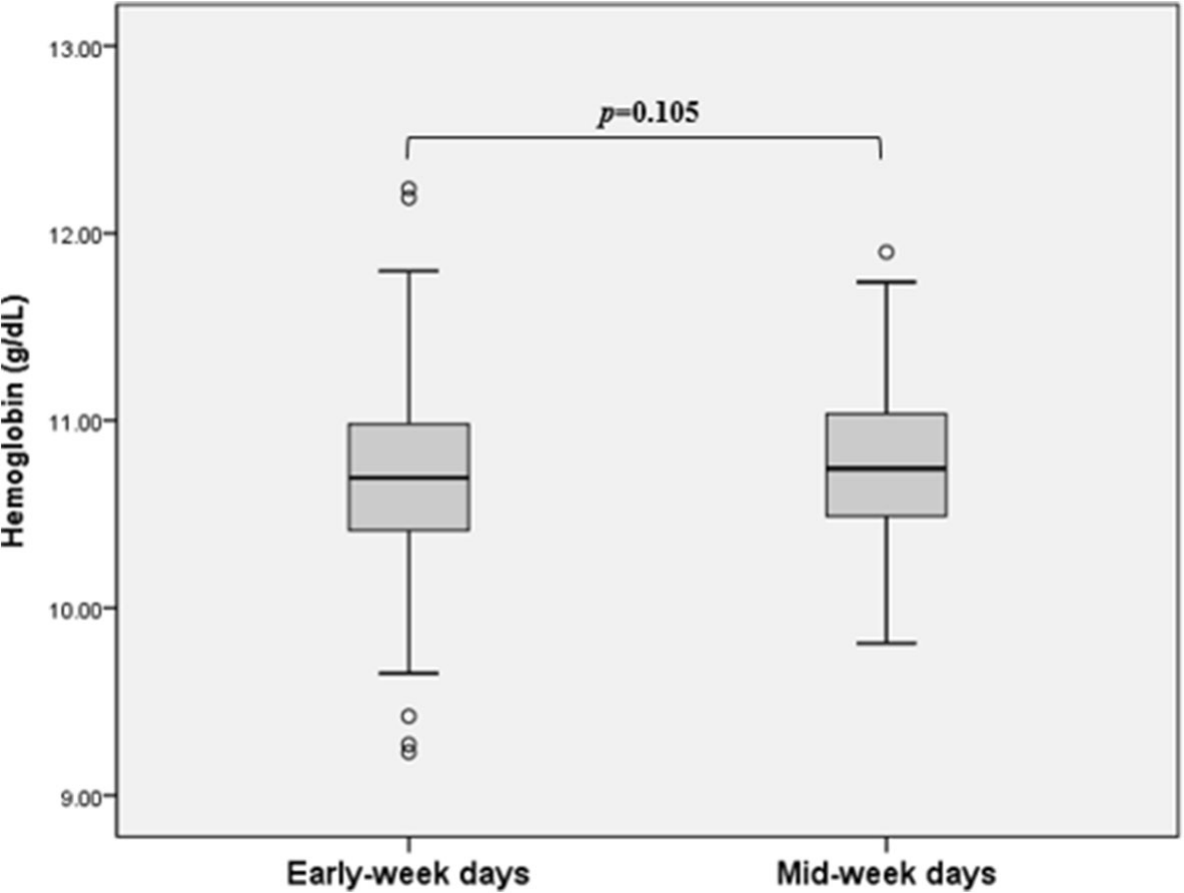
Hemoglobin levels according to hemoglobin measurement day. There was no significant difference in the mean hemoglobin levels between the early-week and mid-week measurements

**Fig. 2:**
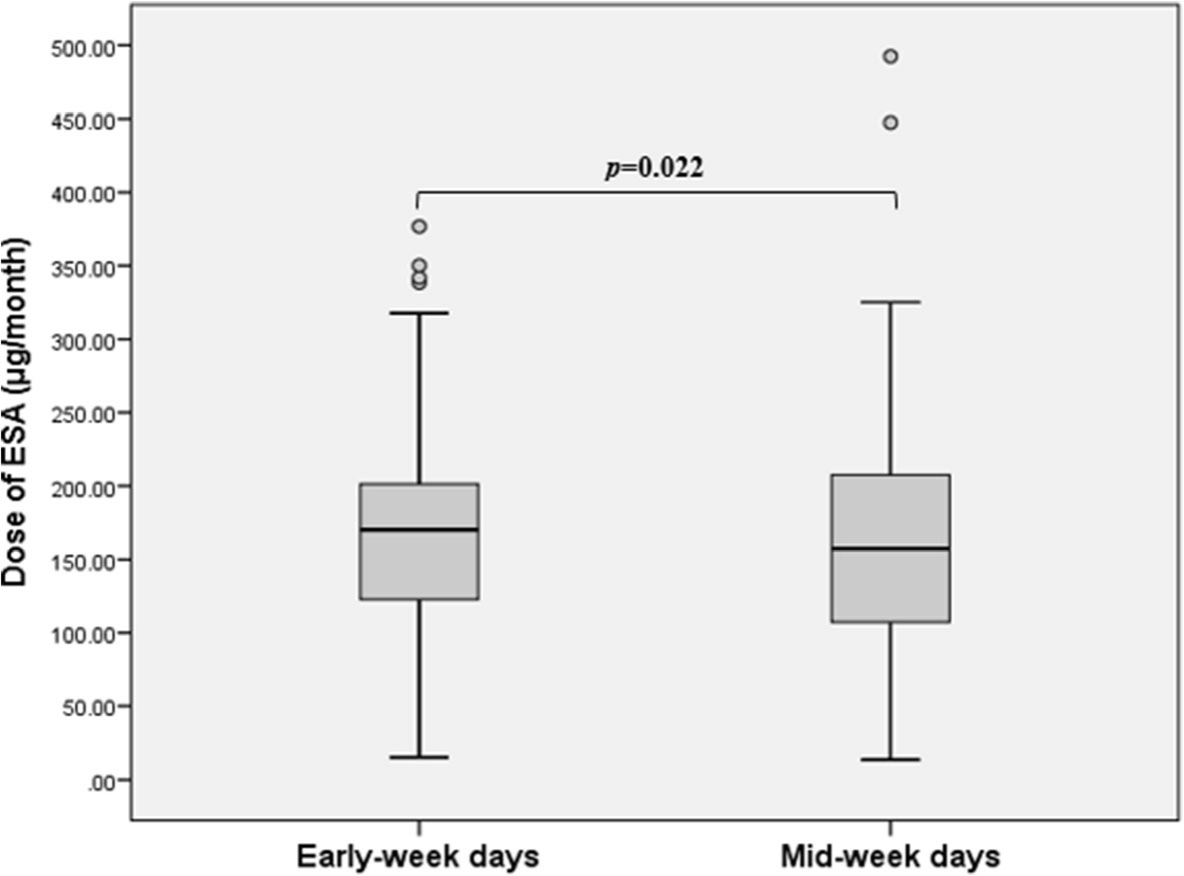
Mean doses of erythropoiesis-stimulating agents before and after changing the hemoglobin measurement day. The mean dose of darbepoetin-α alfa was significantly higher for early-week days than for mid-week days. ESA, erythropoiesis-stimulating agent

Table 2 shows the Hb levels, ESA doses, and parameters related to volume status and anemia according to the Hb measurement days. There was no significant difference in the mean Hb levels between the early-week and mid-week measurements (10.71 ± 0.06 g/dL vs. 10.78 ± 0.47 g/dL, *p* = 0.105) (Fig. 1). However, the mean dose of darbepoetin-α alfa was significantly higher for early-week days than for mid-week days (175.4 ± 72.5 μg/month vs. 163.7 ± 83.6 μg/month, *p* = 0.022) (Fig. 2). Furthermore, the mean IV dose of iron hydroxide sucrose was higher for early-week days than for mid-week days (623.0 ± 489.0 mg/year vs. 447.0 ± 505.2 mg/year, *p* = 0.001).

**Table 2:** Hb levels, ESA and IV iron doses, anemia- and volume status-related parameters

	Early-week days	Mid-week days	*P-*value
Hb (g/dL)	10.71 ± 0.06	10.78 ± 0.47	0.105
Hct (%)	33.83 ± 12.36	32.36 ± 1.53	0.230
Dose of ESA (μg/month) ^a^	175.4 ± 72.5	163.7 ± 83.6	0.022
Median (range)	170.0 (0–376.7)	157.1 (13.3–492.5)	
Interquartile range	123.5–201.9	108.0–208.8	
Dose of IV iron (mg/year) ^b^	623.0 ± 489.0	447.0 ± 505.2	0.001
Parameters related to volume status			
Interdialytic body weight gain (kg)	2.81 ± 0.82	1.99 ± 0.61	< 0.001
Ultrafiltration volume (kg)	2.65 ± 0.73	2.12 ± 0.66	< 0.001
Pre-dialysis body weight/DBW (%)	104.89 ± 3.42	103.51 ± 1.77	< 0.001
Parameters related to anemia			
spKt/V	1.63 ± 0.24	1.67 ± 0.23	< 0.001
Albumin (g/dL)	3.62 ± 0.74	3.76 ± 0.80	< 0.001
Prealbumin (mg/dL)	34.05 ± 7.01	31.94 ± 6.84	< 0.001
hs-CRP (mg/L)	0.26 ± 0.33	0.28 ± 0.33	0.151
Ferritin (ng/mL)	386.0 ± 292.4	308.1 ± 238.0	< 0.001
TSAT (%)	36.43 ± 9.03	37.46 ± 10.90	0.304
iPTH (pg/mL)	205.1 ± 113.7	230.4 ± 129.9	0.021

Values are given as mean ± standard deviation unless otherwise indicated

^a^ darbepoetin alfa. ^b^ iron hydroxide sucrose

Hb, hemoglobin; Hct, hematocrit; ESA, erythropoiesis-stimulating agent; IV, intravenous; DBW, dry body weight; spKt/V, single pool Kt/V; hs-CRP, high-sensitivity C-reactive protein; TSAT, transferrin saturation; iPTH, intact parathyroid hormone

**Fig. 3:**
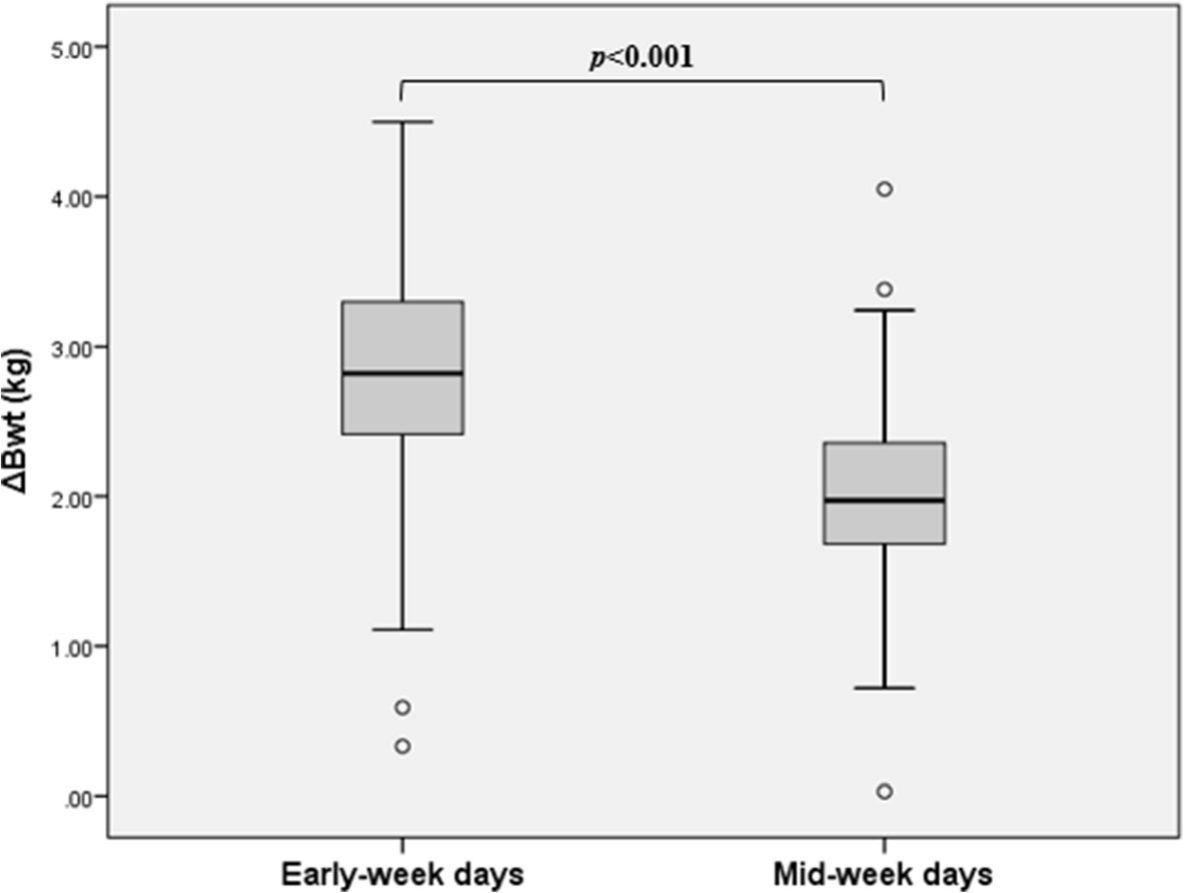
Interdialytic body weight gains according to hemoglobin measurement day. There was a significant difference in the mean interdialytic weight gains between the early-week and mid-week measurements. ΔBwt, interdialytic body weight gain

There was a significant difference in the mean interdialytic weight gains between the early-week and mid-week measurements (2.81 ± 0.82 kg vs. 1.99 ± 0.61 kg, *p* < 0.001) (Fig. 3). There was also a significant difference in the ultrafiltration volumes between the early-week and mid-week measurements (2.65 ± 0.73 kg vs. 2.12 ± 0.66 kg, *p* < 0.001). Furthermore, there was a significant difference in the ratios of pre-dialysis body weight/dry body weight between the early-week and mid-week measurements (104.89 ± 3.42 vs. 103.51 ± 1.77, *p* < 0.001).

Dialysis adequacy was appropriate in both groups, and there were no significant differences between the two groups in terms of high-sensitivity C-reactive protein or transferrin saturation. However, a significant difference in ferritin levels was detected (*p* < 0.001). A significant difference in intact parathyroid hormone levels was also detected (*p* = 0.021), although both levels were within the target range.

Table 3 shows the doses of ESA according to the Hb levels. There was no clinically significant change in the number of patients after changing the Hb measurement day (*p =* 0.125). There was also no significant change in the number of patients with Hb levels of > 10 g/dL (*p =* 0.100). A significantly higher dose of ESA was administrated to patients with lower Hb levels (*p* < 0.001).

**Table 3:** Dose of ESA according to Hb level

	Hb of < 10 g/dL	Hb of 10–11 g/dL	Hb of ≥11 g/dL	*P-*value
Early-week days				
Number of patients	8	64	20	
Hb (g/dL)	9.71 (9.23–9.97)	10.63 (10.03–10.99)	11.45 (11.01–12.24)	
ESA (μg/month)	311.67 (188.33–376.67)	179.17 (89.17–317.50)	93.34 (0–255.83)	< 0.001
Mid-week days				
Number of patients	2	65	25	
Hb (g/dL)	9.88 (9.81–9.94)	10.61 (10.03–10.97)	11.22 (11.0–12.87)	
ESA (μg/month)	292.5 (260–325)	183.33 (69.17–492.50)	95 (13.33–233.33)	< 0.001
*P-*value ^b^	*p* = 0.125 ^a^ (*p* = 0.100 ^b^)			

Values are given as number or median (range)

^a^ Comparing the number of patients for early-week days and mid-week days. ^b^ Comparing the number of patients with Hb levels of > 10.0 g/dL

Hb, hemoglobin; ESA, erythropoiesis-stimulating agent

## Discussion

The present study revealed that mid-week measurements were associated with lower doses of ESA and IV iron, without a difference in Hb levels. The interdialytic weight gain was lower when the measurements were performed on mid-week days. Moreover, there was no significant difference in the number of patients with Hb levels of > 10.0 g/dL, and higher doses of ESA were administered to patients with Hb levels of < 10.0 g/dL than to those with Hb levels of > 10.0 g/dL. It appears that this is the first study to suggest that different doses of ESA may be required based on the day of Hb measurement in clinical practice.

Because short-term Hb variability can be affected by volume status, the measured pre-dialysis Hb levels are higher on mid-week days than on early-week days in HD patients [21]. However, the present study did not reveal a difference in the Hb levels according to the measurement day, although there was a significant difference in the ESA doses. It is possible that changing the Hb measurement day resulted in lower prescribed ESA doses, rather than higher measured Hb levels, because of the reimbursement system in South Korea. In this context, the public medical insurance system covers ESA treatment for CKD anemia up to Hb levels of 11.0 g/dL [29, 30]. In the present study, the median ESA dose before September 2013 was similar to the data from the Korean Clinical Research Center for ESRD (CRC-ESRD), which suggests that mid-week pre-dialysis Hb measurements could result in lower prescribed ESA doses. In addition, patients with lower Hb levels received higher ESA doses in the present study, which reflects target-based ESA dosing because patients with ESA hypo-responsiveness require a higher ESA dose to achieve their target Hb level. This trend has also been observed during the secondary analysis of data from randomized controlled trials (i.e., the CHOIR and TREAT trials) [13, 14].

Because of short-term Hb variability, several discussions addressed the estimation of a representative Hb level in ESRD patients receiving HD [21, 24, 26]. Bellizzi et al. first recommended identifying anemia based on the pre-dialysis Hb level after a short interdialytic period (i.e., mid-week days) [21]. Krisper et al. proposed using the Hb time-averaged concentration (Hb-tac) and developed a formula to calculate Hb-tac using the pre- and post-dialysis Hb levels [24]. Other studies estimated the required dose of ESA based on values other than the pre-dialysis Hb level [25, 26]. Siga et al. reported that the estimated ESA doses based on Hb-tac or the average of pre- and post-dialysis Hb levels were significantly lower than the dose estimated based on the mid-week pre-dialysis Hb level [26]. Castillo et al. also reported a significantly lower estimated ESA dose based on the post-dialysis Hb level [25]. These findings suggest that the recommended ESA dose can vary according to the timing and/or method for determining the representative Hb level in HD patients.

The published guidelines for CKD anemia do not definitively address Hb measurement days and target Hb levels for ESA therapy. Based on varying levels of evidence, most Western guidelines prefer mid-week pre-dialysis sampling for Hb measurement, with a target Hb range of 11.0–12.0 g/dL, regardless of renal replacement therapy modality. For example, the KDOQI guidelines recommended an Hb target of 11.0–12.0 g/dL and mentioned that mid-week pre-dialysis sampling is theoretically optimal, although they acknowledge that pre-dialysis sampling can be performed without a preference for specific days of the week, based on a lack of data regarding patient outcomes according to sampling timing [1, 17]. The KDIGO guidelines also generally recommended an Hb target of 11.5 g/dL and mentioned that Hb monitoring is traditionally performed before a mid-week HD session to minimize Hb variability, although they acknowledge that the mid-week timing is not essential [18]. The Canadian Society of Nephrology guidelines also recommended an Hb target of 11.0 g/dL and indicated that mid-week pre-dialysis Hb measurement is preferable because of Hb variability and a lack of association between post-dialysis Hb levels and clinical outcomes (Grade D recommendation) [27, 31]. In contrast, the Japanese Society for Dialysis Therapy (JSDT) guidelines recommended pre-dialysis Hb measurement at the beginning of the week (2 days from the last dialysis session) with an Hb target of 10.0–11.0 g/dL (moderately strong recommendation). The JSDT recommendation is based on the prevalent Japanese practice of blood sampling at the beginning of the week, as well as studies regarding the survival of Japanese maintenance HD patients by Hirasawa et al. and the JSDT. In a recent study by the Korean CRC-ESRD, Hb levels of 10.0–11.0 g/dL were found to provide a survival benefit among Korean HD patients, although that study did not consider the day or timing of the Hb measurement. Furthermore, the Korean CRC-ESRD study revealed that patients with Hb levels of ≥10.0 g/dL and a median ESA dose of < 126 μg/week/kg had the best survival probability [29].

In the present study, a lower ESA dose was observed after the change in the day of the Hb measurement. From the perspective of Hb variability, a change in the Hb measurement day might actually result in a decreased target Hb level. Given the price of Nesp® (about $17.3 per 20 mcg prefilled syringe in South Korea, April 2019), the number of ESRD patients in South Korea in the year of 2017 (73059), and the approximate difference of darbepoetin alpha in our study (10 mcg / month/person), the cost difference would be at least $ 7 million a year in South Korea. Considering the huge cost and that the administered ESA dose and the achieved Hb level both are critical prognostic factors, it is important to properly determine the representative Hb level in ESRD patients receiving HD. Because anemia in ESRD patients can cause poor outcomes, including shortened survival, ESA dosing based on early-week Hb measurements may be desirable. However, because of the short-term variability in Hb levels, ESA dosing based on mid-week Hb measurements with a higher target Hb level may be more accurate. Moreover, considering the complications and poor prognosis related to a higher ESA dose and/or ESA hypo-responsiveness, ESA dosing based on mid-week Hb measurement with the current Hb target level may be an effective approach. Therefore, a clinical trial with pre-dialysis Hb measurement on mid-week days is likely needed to precisely identify the target Hb level needed to improve the prognosis of HD patients. Future studies should also consider patient ethnicity, given that there are ethnic differences in Hb levels.

The present study has some limitations. First, sample size is small considering the small difference of ESA dose before and after the change of Hb measurement day. Despite the small sample size, considering the scarcity of study about Hb measurement day in clinical practice and the large number of ESRD patients and burden worldwide, we think this study is still meaningful. Future studies about CKD anemia should take account of the Hb measurement day. Second, there are several study design limitations, such as selection bias, information bias and confounding. The physicians who prescribed the ESA were changed annually in our center, which suggests that inter-individual differences in ESA dosing practice could have influenced the results. Third, the study did not evaluate clinical outcomes related to the ESA dose. Echocardiographic parameters such as left ventricular ejection fraction, left ventricular mass index would be important parameters related to the outcomes, but only few patients underwent echocardiography twice during the study period.

## Conclusion

In conclusion, relative to early-week measurements, mid-week pre-dialysis Hb measurements were significantly associated with lower doses of ESA and IV iron to maintain the target Hb level in ESRD patients receiving HD. Thus, mid-week Hb measurements would be a better criterion for determining the appropriate ESA dose.

## Data Availability

The datasets used and/or analyzed during the current study are available from the corresponding author on reasonable request.

## Abbreviations

CKD: Chronic kidney disease
CRC-ESRD: The Korean clinical research center for end stage renal disease
CVA: Cerebrovascular accident
CVD: Cardiovascular disease
EPO: Erythropoietin
ESA: Erythropoiesis-stimulating agent
ESRD: End stage renal disease
Hb: Hemoglobin
Hct: Hematocrit
HD: Hemodialysis
IV: Intravenous
JSDT: Japanese Society for Dialysis Therapy
KDIGO: The Kidney Disease Improving Global Outcomes
KDOQI: The National Kidney Foundation Kidney Disease Outcomes Quality Initiative
rhEPO: Recombinant human erythropoietin
RRT: Renal replacement therapy
